# Laser doppler flowmetry to detect pulp vitality, clinical reference range and coincidence rate for pulpal blood flow in permanent maxillary incisors in Chinese children: a clinical study

**DOI:** 10.1186/s12903-023-02747-z

**Published:** 2023-05-12

**Authors:** Kuan Yang, Feifei Guo, Zhifei Zhou, Zeming Hui, Zirui Wang, Junhui Wang, Yujiang Chen, Xin Ge, Ruizhe Huang, Xiaojing Wang

**Affiliations:** 1 Department of Orthodontics, College of Stomatology, Xi’an Jiaotong University, Xi’an, China; 2 Department of Orthodontics, Affiliated Hospital of Qingdao University, School of Stomatology, Qingdao University, Qingdao, China; 3 State Key Laboratory of Military Stomatology and National Clinical Research Center for Oral Diseases and Shanxi Key Laboratory of Stomatology, Department of Pediatric Dentistry, Fourth Military Medical University, Xi’an, 710032 China; 4 Key laboratory of Shaanxi Province for Craniofacial Precision Medicine Research，College of Stomatology, Xi’an Jiaotong University, Xi’an, China

**Keywords:** Clinical reference range, Clinical coincidence rate, Laser doppler, Pulp vitality, Pulpal blood flow

## Abstract

**Background:**

A laser doppler flowmetry (LDF) test can reflect the pulp vitality caused by the change in pulp blood flow (PBF). This study aimed to investigate the PBF of the permanent maxillary incisors using LDF and to calculate the clinical reference range and coincidence rate for pulp vitality using PBF as an indicator.

**Methods:**

School-age children (7–12 years) were recruited randomly. A total of 455 children (216 female and 239 male) were included in this study. An additional 395 children (7–12 years) who attended the department due to anterior tooth trauma from October 2015 to February 2018 were included to assess the clinical occurrence rate. The PBF was measured using LDF equipment and an LDF probe.

**Results:**

The clinical reference range of PBF values for the permanent maxillary incisors (teeth 11, 12, 21, and 22) in children were from 7 to 14 perfusion units (PU), 11 (6.016; 11.900 PU), 12 (6.677; 14.129 PU), 21 (6.043;11.899 PU), and 22 (6.668; 14.174 PU). There was a statistically significant correlation between PBF and children’s age (*p* < 0.000) without any significant gender discrimination (*p* = 0.395). For all incisors, for any age group, the PBF detection value of the lateral incisors was significantly higher than that of the central incisors (*p* < 0.05). The clinical coincidence rate of detecting PBF in the traumatic teeth was 90.42% and the sensitivity and specificity were 36.99% and 99.88%, respectively.

**Conclusions:**

The determination of the PBF clinical reference range and clinical coincidence rate for the permanent maxillary incisors in children using LDF provided a promising theoretical basis for clinical applications.

## Background

Dental trauma is a common problem that is challenging in dental diagnosis and treatment. The diagnosis and treatment plan depends mainly on the accurate judgment of the state of the dental pulp [1]. Accurate detection of dental pulp vitality is conducive to preserving dental pulp functionality and promoting the development of tooth roots in young children [2]. The common methods used to detect pulp vitality in clinical practice are thermal and electrical pulp testing (EPT) [3]. However, due to the open apex, young permanent teeth cannot form a high-resistance loop [4]. Similarly, the early stages of dental trauma [5] lead to false results or no reaction to the sensory test methods due to the shock phase. In addition, children's coordination is poor due to their high sensitivity to painful stimuli, especially after tooth trauma. The inaccurate judgment of the dental pulp condition poses a significant challenge to diagnosis. The undiagnosed inflammation or necrosis of dental pulp might cause certain complications, which include apical periodontitis, tooth loosening, and even tooth loss [6].

Extensive research has been conducted, which proposed a new concept where the decisive factor that influenced pulp vitality was blood circulation rather than the nervous system [7]. Laser Doppler flowmetry (LDF) uses the helium–neon or semiconductor diode as the laser light source and the reflected light from frequency drift caused by the movement of blood cells according to the doppler phenomenon is absorbed. The changes in the flow and flow rate of the blood cells according to the intensity and the frequency drift are detected [8]. The test object of LDF is the local microcirculation system of the teeth [9], which could reflect the state of pulp vitality due to the changes in pulpal blood flow (PBF). LDF is noninvasive, painless, objective, convenient, and non-radioactive [10], which effectively avoids a pseudo-response caused by nerve shock or hypersensitivity [11]. Therefore, there is a wide range of potential applications in children's dental treatment [12].

LDF has no standard clinical reference range for dental pulp (i.e., a physiological range of healthy and vital pulp) [12]. Many factors might affect establishing a reference range for pulp vitality measured by LDF, such as the consistency of the detection, the accuracy of different types of instruments, the anti-interference performance of the instrument, and a lack of standardized detection methods. The study aimed to investigate the clinical reference range of PBF in the permanent maxillary incisors using LDF, which could be an indicator of pulpal health in children.

## Methods

### Objects

This study was approved by the institutional research ethics committee at the Stomatological Hospital of the Fourth Military Medical University, China (Approval number: IRB-REV-2016044).

School-age children (7–12 years old; n = 455) with healthy teeth were randomly recruited according to the preset selection criteria as mentioned in the following section. An additional number of school-age children (7–12 years old; n = 355) who attended the department due to trauma to the anterior teeth were also included. The inclusion and exclusion criteria of this study were formulated according to the application precautions for LDF. The target population that met the experimental requirements was screened. The subjects and their guardians were informed of the experimental methods and aims. All the recruited participants were willing to participate in the study and a written informed consent was obtained from their guardians. This study was conducted following the guidelines of the Declaration of Helsinki.

### Selection criteria

Two pediatric stomatologists (JW, and YC) performed the clinical examination and intraoral periapical radiographic evaluation to determine the following inclusion and exclusion criteria, The inclusion criteria for normal healthy children's teeth included: (1) the target teeth should have the required eruption height (the occlusal surface should be ≥ 5 mm above the gingival margin); (2) good oral hygiene and periodontal health; (3) teeth were free from pigmentation, caries, or malocclusions, with no history of anterior tooth trauma, restorative, or orthodontic treatment; (4) a good state of general health without a history of any systematic, infectious, or genetic diseases; and (5) cooperative when performing the test.

The exclusion criteria for normal healthy children's teeth included: (1) any subjects that were sensitive to a silicone rubber impression material; (2) they suffered from any hematological or other systemic illnesses; and (3) they had used cardiovascular drugs within the previous 6 months.

The inclusion criteria for traumatic teeth included: (1) a clear diagnosis of traumatic simple crown fractures and tooth luxation, uncomplicated crown fracture, crown–root fracture, and root fracture; and (2) they met the inclusion criteria for normal healthy teeth.

The exclusion criteria for traumatic teeth included: (1) the participants with avulsed teeth, had decapitated, or the section of traumatic teeth was < 3 mm from the marginal gingiva; (2) undergoing traumatic tooth fixation or orthodontic treatment; and (3) meeting the exclusion criteria for normal healthy teeth.

### Sample size calculation

According to the inclusion criteria for healthy children mentioned previously, 40 children were selected for pre-experiments, which included 20 males and 20 females, with an average age of 8.97 ± 1.26 years. The measured PBF value was 9.62 ± 1.96. Therefore, the actual sample size was 410. Considering 10% (41) was the number of lost visits, 451 participants were determined to achieve the required power (80%) and significance level (95%). Similarly, for children with dental trauma, 58 children were selected for pre-experiments, including 35 males and 23 females, with an average age of 9.08 ± 1.88 years. The measured PBF value was 9.08 ± 2.54. Therefore, the actual sample size was 355. Considering 10% (36) was the number of lost visits, 391 participants were determined to achieve the required power (80%) and significance level (95%). Two pediatric stomatologists (JW, and YC) performed the LDF measurements. The kappa values of both stomatologists were 0.84 and 0.86, respectively and the kappa value for consistency between both stomatologists was 0.82. Because the consistency was relatively good, one stomatologist (JW) performed all the formal LDF measurements. In case of differences between the three measurements, a second surveyor (YC) was involved to verify the measurement results. Follow-up visits were scheduled at the initial visit, 1, 3, 6, and 12 months after surgery and pulp viability was evaluated through PBF data acquisition.

### PBF data acquisition

**Fig. 1:**
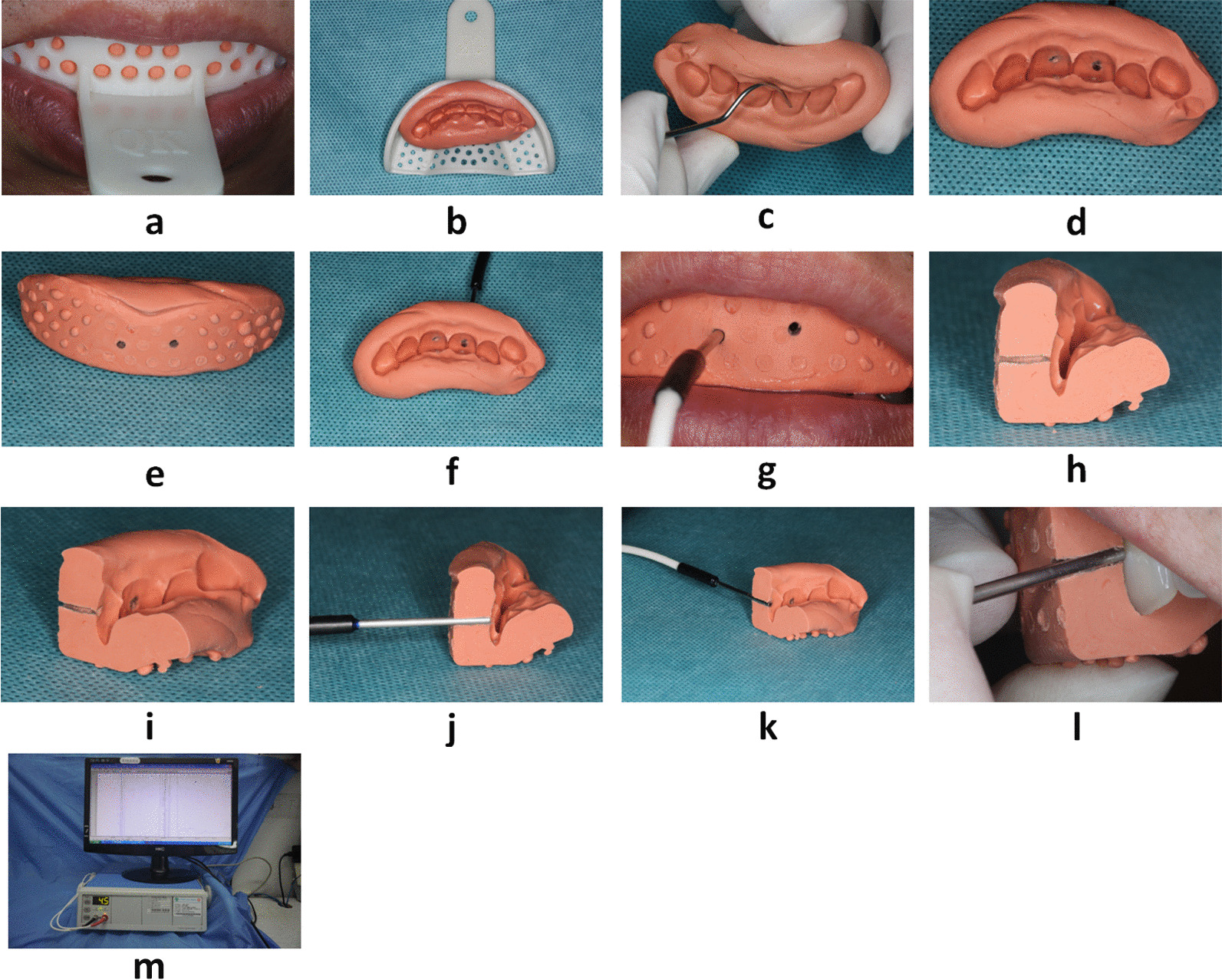
Presentation of the detection process that used a doppler blood flow tester: **a** preparation of silicone rubber impression of upper anterior teeth of patients that used heavy body impression putty; **b** silicone rubber impression preparation removed from the oral cavity and disinfected; **c** marking the anatomical location for probing; (**d** and **e**) lingual and labial views after drilling the holes; **f** test probe trimming and punching position; **g** placement of the test probe; **h** longitudinal section of silicon rubber offset printing die after cutting; **i** view of the lingual surface of the longitudinal section after the silicon rubber offset printing die is cut; (**j** and **k**) probe placement after longitudinal sectioning the silicon rubber offset die; (**l**) placement of the probe in the sectioned silicone rubber impression when testing in the oral cavity; (**m**) LDF equipment in the clinical settings

To unify the standard, all data collected in this study were taken at room temperature (20 °C) and the subjects were maintained in a seated and calm state. A silicone rubber impression (3 M ExpressTMSTD; 3 M ESPE Dental Products, Minnesota, USA) was made for the subjects after the maxillary anterior testing area was cleaned thoroughly. The thickness of the labial side of the impression was maintained at approximately 3–5 mm; therefore, effective retention for the probe could be formed to enhance the stability of the detection after punching. Corresponding to the teeth under investigation, the position of drilling in the impression was situated in the middle of the tooth surface and approximately 3–5 mm from the gingival margin [13]. The diameter of the drilling hole was consistent with the probe. The diameter of the drilling hole was consistent with the LDF probe (diameter; 1.6 mm, 785 nm: Perimed, DP 416, Perimed AB, Stockholm, Sweden). The laser Doppler blood flow tester was connected and warmed up for 10 min. The LDF system was debugged and then calibrated the value with the standardization liquid (Pf 1000, Sweden). The silicone rubber impression was reset to the detected area for testing (Fig. 1 [14]). Test subjects were asked to abstain from hot or cold foods and beverages before attending for LDF measurements (Perimed, PF 5001, Perimed AB; Stockholm, Sweden) and to maintain normal and steady breaths and keep their head static during LDF measurements to ensure the stability of the detected data. In general, the time for detection was 1–3 min after which the output waveform was relatively stable, the test results were taken from a relatively stable waveband and the required time was ≥ 30 s and the standard deviation (SD) < 4 in the output result was regarded as a valid testing result. The PBF of the maxillary incisor teeth was detected following this step and the test results were recorded as mentioned previously. The subjects were asked to have a quiet rest for 10 min after the test was completed and then the previous test was repeated, and the corresponding record was recorded as the second measurement. The mean value of both measurements was calculated for further data interpretation. Finally, photographs and intraoral periapical radiographs of the maxillary anterior teeth were taken.

### Diagnostic criteria for clinical compliance rate in traumatic teeth

The LDF test results of the affected teeth were compared with the range of clinical reference values (which were derived from the data from the test statistics of healthy children). The PBF of traumatic, contralateral homonymous, and adjacent teeth were measured and compared with the data from the normal reference values and the clinical imaging test results. The EPT (Pulp vitality tester, DY310, DENJOY DENTAL CO, LTD, China) results were considered for the affected teeth with completed root development. Combined with the precautions in the application of LDF, the corresponding positive and negative diagnostic criteria were formulated.

Negative criteria: it was recorded as negative if the results suggested that the patients with dead pulp should be verified by diagnostic pulpotomy, and the pulpotomy confirmed the pulp necrosis. In contrast, confirmation of vital pulp through diagnostic pulpotomy was recorded as a false negative.

Positive criteria: if the results suggested that the patients with live pulp should be followed-up long-term. During the visit, no pathological changes in the affected teeth were recorded as positive. In contrast, pulp necrosis in a short time (< 2 weeks) was recorded as a false positive.

### Statistical analysis

The test results were statistically analyzed using SPSS (V 22.0) software (IBM, Armonk, NY, USA). The basic characteristics of the subjects were described for age and gender. The mean, standard deviation, and reference range of the subjects’ PBF values were described based on their age, gender, and tooth type. The least significant difference (LSD) t-test and multi-factor analysis of variance (ANOVA) were implemented, which included age, gender, and tooth type. The mean values of multiple samples were compared using the LSD t-test to analyze whether there was a statistical difference between the average number of multiple samples. Statistical analysis was performed on the root development of the maxillary central and lateral incisors in the children. The clinical coincidence rate of LDF in detecting PBF in traumatic teeth was calculated according to the four-grid table and sorted according to the evaluation data of the diagnostic test. The test level α = 0.05.

## Results

### Sample information

According to the inclusion and exclusion criteria, 455 (e.g., male 239 and female 216) healthy subjects were included in this study, the average ages were 8.65 ± 0.94 years. The age distribution of the sample population is shown in Table 1. In total, 909 central incisors and 785 lateral incisors met the test criteria (some of the subjects had no lateral incisors or the eruption height did not meet the requirement for detection). In total, 395 traumatized children (251 male and 144 female) and 550 traumatized teeth (349 male and 201 female) were included in this study, the average ages were 9.86 ± 0.76 years. In the simple crown fracture group, 199 children (132 males and 67 females) had 264 teeth (180 males and 84 females); In the dental concussion group, 103 children (60 males and 43 females) had 158 teeth (87 males and 71 females); In the tooth dislocation group (subluxation, lateral displacement, and setback), 93 children (59 males and 34 females) had 128 teeth (82 males and 46 females). All the participants were fully cooperative, and no child was excluded due to the uncooperative behavior.

**Table 1:** General characteristics of the sample population (*n* = 455)

	*n*	%
*Gender*		
Male	239	52.53
Female	216	47.47
*Age (years)*		
7	95	20.88
8	104	22.86
9	127	27.91
10	67	14.73
11	38	8.35
12	24	5.27

### Clinical reference range

The PBF detection values of maxillary incisor teeth in different gender samples were statistically described and the clinical reference range for the PBF in the anterior teeth in children aged 7–12 years was calculated (Tables 2, 3, 4). The clinical reference range for PBF values for the anterior teeth in children was from 7 to 14 perfusion units (PU), 11 (6.016; 11.900 PU), 12 (6.677; 14.129 PU), 21 (6.043;11.899 PU), 22 (6.668; 14.174 PU). For all incisors, for any age group, the PBF detection value for lateral incisors was significantly higher than that of central incisors (*p* < 0.05).

**Table 2:** PBF values (PU) of maxillary anterior teeth in females of different ages

Age (years)	Tooth type											
	12			11			21			22		
	Mean	SD	Reference range	Mean	SD	Reference range	Mean	SD	Reference range	Mean	SD	Reference range
7	9.94	2.13	(5.76, 14.11)	8.56	1.49	(5.64, 11.48)	8.59	1.61	(5.44, 11.73)	9.74	1.62	(6.57, 12.91)
8	10.38	1.78	(6.89, 13.86)	8.77	1.42	(5.98, 11.55)	8.68	1.68	(5.38, 11.97)	10.25	1.85	(6.62, 13.88)
9	10.66	2.00	(6.73, 14.58)	9.14	1.50	(6.20, 12.08)	9.06	1.40	(6.31, 11.81)	10.82	1.79	(7.30, 14.33)
10	10.78	1.87	(7.12, 14.45)	9.27	1.66	(6.02, 12.53)	9.31	1.58	(6.22, 12.41)	10.41	1.93	(6.63, 14.20)
11	11.50	1.99	(7.61, 15.40)	9.26	1.40	(6.51, 12.01)	9.48	1.59	(6.36, 12.59)	11.26	1.93	(7.48, 15.05)
12	9.79	2.94	(5.12, 14.47)	8.25	1.12	(6.47, 10.03)	7.83	1.04	(6.18, 9.48)	9.26	2.08	(5.95, 12.57)

**Table 3:** PBF values (PU) of maxillary anterior teeth in males of different ages

Age (years)	Tooth type											
	12			11			21			22		
	Mean	SD	Reference range	Mean	SD	Reference range	Mean	SD	Reference range	Mean	SD	Reference range
7	8.67	1.16	(6.39, 10.95)	8.52	1.41	(5.75, 11.29)	8.54	1.27	(6.04, 11.03)	8.85	1.11	(6.67, 11.03)
8	9.93	1.62	(6.76, 13.10)	8.99	1.62	(5.82, 12.15)	8.99	1.56	(5.94, 12.04)	9.97	2.03	(5.99, 13.94)
9	10.47	1.90	(6.76, 14.19)	8.95	1.48	(6.05, 11.85)	9.05	1.27	(6.57, 11.54)	10.62	1.82	(7.05, 14.18)
10	10.87	1.94	(7.08, 14.66)	9.38	1.52	(6.40, 12.37)	9.49	1.62	(6.31, 12.67)	11.04	2.01	(7.09, 14.99)
11	10.54	1.47	(7.65, 13.42)	8.91	1.17	(6.61, 11.20)	8.93	1.27	(6.44, 11.42)	10.56	1.78	(7.06, 14.05)
12	11.56	1.52	(10.28, 12.83)	9.14	1.52	(7.87, 10.41)	9.12	0.89	(8.37, 9.86)	11.26	1.91	(9.66, 12.85)

**Table 4:** PBF values (PU) of maxillary anterior teeth in children of different ages

Age (years)	Tooth type											
	12			11			21			22		
	Mean	SD	Reference range	Mean	SD	Reference range	Mean	SD	Reference range	Mean	SD	Reference range
7	9.34	1.83	(5.74, 12.93)	8.54	1.41	(5.77, 11.31)	8.57	1.27	(6.07, 11.06)	9.32	1.11	(7.14, 11.50)
8	10.07	1.62	(6.90, 13.24)	8.90	1.62	(5.74, 12.07)	8.86	1.56	(5.81, 11.90)	10.06	2.03	(6.08, 14.04)
9	10.53	1.90	(6.82, 14.25)	9.03	1.48	(6.12, 11.93)	9.04	1.27	(6.56, 11.53)	10.69	1.82	(7.12, 14.25)
10	10.79	1.94	(6.99, 14.58)	9.32	1.52	(6.34, 12.31)	9.44	1.62	(6.25, 12.62)	10.77	2.01	(6.82, 14.72)
11	11.12	1.47	(8.24, 14.00)	9.14	1.17	(6.85, 11.44)	9.31	1.27	(6.82, 11.79)	10.97	1.78	(7.48, 14.47)
12	10.97	2.14	(9.61, 12.33)	8.84	1.41	(7.95, 9.74)	8.69	1.09	(7.99, 9.38)	10.59	2.11	(9.25, 11.93)
Total	10.40	1.901	(6.677, 14.129)	8.96	1.501	(6.016, 11.900)	8.97	1.494	(6.043, 11.899)	10.42	1,915	(6.668, 14.174)

### Factors that affect PBF

**Fig. 2:**
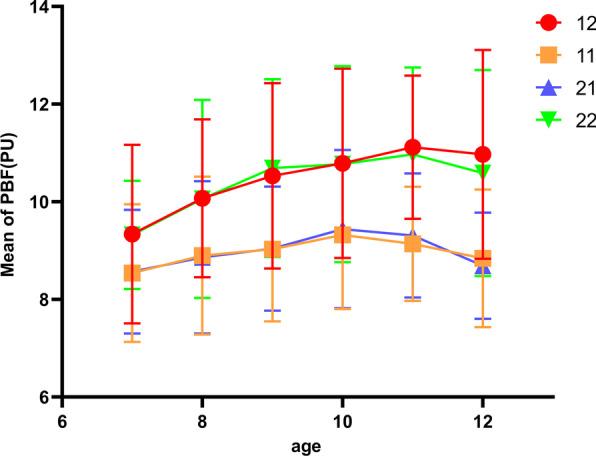
Trend in PBF value in children of different ages, which shows the trend in the PBF of 12/11/21/22 teeth with age and that the PBF gradually increased with age and then tended to be stable or decreased slightly

**Fig. 3:**
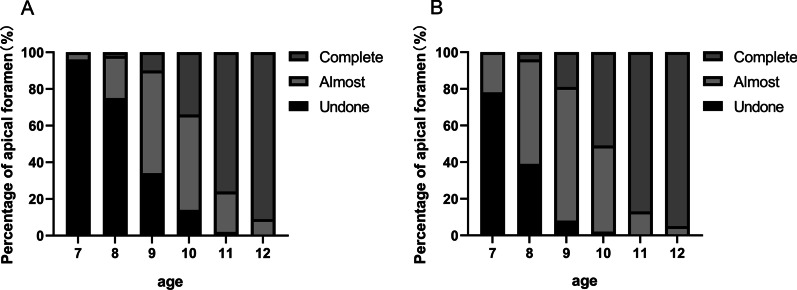
Apical foramen of central and lateral incisors in children of different ages, which shows the changes in root development of incisors with age and the individual differences in each age were obvious regardless of: **a** central incisors; and **b** or lateral incisors

A multi-factor analysis of variance was performed on the PBF data of all study subjects (Table 5). The results showed that the difference in PBF values for children's permanent maxillary incisors between genders was not statistically significant (*p* = 0.395). In contrast, the mean PBF exhibited significant differences for participants’ ages (*p* < 0.001) and types of incisors. PBF values for different tooth types were different. Specifically, the PBF differences between teeth 11 and 21, and teeth 12 and 22 were not statistically significant (*p*˃0.05), and the PBF value of the central incisors was lower than that of the lateral incisors. The interaction effect between gender and age was significant (*p* < 0.01). Between the sample populations of different genders, age factors had different effects on PBF values (Fig. 2). The PBF detection value in young permanent teeth was significantly correlated with age, and there was a certain relationship between the development of tooth root and age (Fig. 3); therefore, PBF might be closely related to the degree of root development.

**Table 5:** Multivariate ANOVA values of LDF

Factor	Type III sum of squares	df	Mean square	*F*	*p-*value
Correction model	1196.650	47	25.461	9.172	< 0.0001
Intercept	108,963.355	1	108,963.355	39,252.815	< 0.0001
Gender	2.014	1	2.014	0.725	0.395
Age	210.227	5	42.045	15.146	< 0.0001
Tooth type	539.876	3	179.959	64.828	< 0.0001
Gender * age	48.140	5	9.628	3.468	0.004
Gender * tooth type	5.743	3	1.914	0.690	0.558
Age * tooth type	39.010	15	2.601	0.937	0.522
Gender * age * tooth type	22.799	15	1.520	0.548	0.914
Error	4569.193	1646	2.776		
Total	163,071.192	1694			
Total number of post-corrections	5765.842	1693

### PBF, age, and root development

The distribution of tooth root development is shown in Fig. 3. According to Nolla's tooth development stage [15], root development can be divided into various stages, such as the completion of one-third or two-thirds of the root, almost complete root development with an open apex, and complete root development. The results showed that the degree of individual development of tooth roots in children was near completion (open apex). More than 50% of the central and lateral incisors were completely developed at 10 and 11 years old, respectively. Based on the evaluation that used the intraoral periapical radiographs, the root development of the lateral incisors lagged behind the central incisors. In addition, the root canal diameters of lateral incisors at the same age were larger than those in the central incisors at any root level. This individual difference might be why there was a corresponding trend in PBF with age and a difference between PBFs in the central and lateral incisors.

### Clinical coincidence rate of LDF in detecting PBF of traumatic teeth

The clinical coincidence rate of using LDF to detect PBF was 90.42%, While the sensitivity was 36.99%, and the specificity was 99.88%. (Table 6).

**Table 6:** Clinical coincidence rate of LDF when detecting PBF of traumatic teeth

Diagnostic experiment	Standard diagnosis		Total
	Negative	Positive	
Negative	54 (36.98%)	92 (63.02%)	146
Positive	1 (0.12%)	824 (99.88%)	825
Total	55 (5.66%)	916 (94.34%)	971

## Discussion

The present study investigated the pulp vitality and PBF of the permanent maxillary incisors in healthy children using LDF. The range of the PBF for clinical reference for healthy permanent anterior teeth was determined in children from different age groups. In addition, this study considered various factors that might affect the PBF, such as gender, tooth type, root development and age [16].

For gender, there were no statistically significant differences between all the age groups. The LDF evaluates the changes in blood flow velocity in the pulp capillaries that might change under certain systemic conditions, such as the body's blood pressure and heart rate [17]. Therefore, the detection of pulp vitality by LDF is not suitable for patients with hypertension, obvious anemia, and other hematological systemic diseases [18]. However, these findings might differ in other ethnic groups. A study [19] showed that the mean blood pressure in young women was slightly higher than the mean among children aged 7–12 years old. Therefore, the range of clinical reference for the anterior teeth PBF could potentially be the same for males and females in the study group.

The present study reported a statistically significant difference in the PBF detection value between the maxillary central and lateral incisors of the same gender. The PBF detection value for the lateral incisor was slightly higher than that of the central incisors. This could be due to the changes in the shape of the anterior pulp cavity with age. From the root canal development with the trend in the age that the root development of the lateral incisors was behind the central incisors and the pulp cavity was in a more open state. The larger the volume of the pulp cavity, the more the pulp blood flow will be in unit time. However, the volume of the central incisors increased more than the lateral incisors with a gradual development of the root and closure of apical foramen in adolescents, which could change the PBF trend. The results demonstrated higher PBF values with a higher proportion of trumpet shapes; however, this trend of a gradual increase stabilized, followed by a slight decline with age. Ikawa et al. [20] reported contrasting findings that might be attributed to several reasons. First, the sample size included in their research was relatively small. Second, the age span was relatively large, and the number of teenagers and children was relatively small. A Laser Doppler blood flow tester reflects the state of the dental pulp by detecting the flow and velocity of blood cells [8]. The main reason for the contrasting result could be attributed to the complete development of the root apex of the anterior teeth after the age of 11–12 years, In addition, the shape and volume of the pulp cavity and the blood supply gradually tended to be stable after the age of 11–12 years, and before the age of 11 years, most of the roots are not fully developed, the apical foramen is not closed, and the blood flow in the pulp cavity becomes larger. The age of children in this study group was 7–12 years. The root was still in the development stage or near the completion stage of development (open apex). Therefore, the blood flow in the pulp cavity tended to change significantly.

A comprehensive analysis of this phenomenon might be related to the eruption height of the teeth and the location in the gums. In young children, the eruption of teeth is a dynamic process that alters the proportion of erupted teeth in the occlusal line and the cervical margins of the gingiva with time. Specifically, the eruption height gradually increases, and the gingival margins gradually recede with age. Therefore, the LDF probe positioning on the teeth changes, which leads to a decrease in the amount of laser light transmitted into the marrow cavity [13]. This is one of the main limitations when this method is used for partially erupted teeth. However, due to the continuous eruption of the teeth and the gradual retraction of the gingival position, the position of the probe gradually reached a standard testing position and the amount of laser light transmitted into the pulp cavity increased. This increased the doppler shift, which resulted in the phenomenon where the PBF detection value showed a gradually increasing trend [17]. However, the blood flow into the pulp cavity decreases gradually after root development and the doppler shift generated by the same laser transmission decreases [1]. However, most of the roots were completely developed in the curve of PBF with age. Therefore, the factors that affected the PBF in children could be the local microcirculation system of the tooth, the degree of root development, the location of the gingiva, and other comprehensive factors.

To verify the reliability of the reference range and provide a reference for further research and clinical applications, the clinical coincidence rate was tested using the LDF to evaluate the pulp vitality of traumatic teeth. The results of the present experimental study revealed that LDF has a high clinical coincidence rate (˃ 90%) in detecting the pulp vitality of traumatized maxillary teeth. LDF detection exhibited a high specificity but low sensitivity. This was because the previously discussed phenomenon might be attributed to either a temporary reversible reduction of medullary blood supply caused by tooth trauma or the interference of therapeutic materials on the transmission of the laser. In addition to the previous factors, the mental state of the subjects might have affected the results. For example, when the subjects were excessively tired, sleepy or had a high-grade fever, the PBF test value might have been reduced to a certain extent, which resulted in false positive results. In contrast, the increase in blood pressure during the test might have affected the accuracy of the test results and obscured the existing problems. Therefore, during clinical testing, attention must be paid to the physical and psychological conditions of the patients and assist them with comfortable and relaxed seating.

The use of LDF had some limitations and clinically uncontrollable factors [21], such as the individual differences between PBF and, different optical characteristics of tooth structures, ethnic differences, tooth morphology, and periodontal tissue. Similarly, technical parameters, such as LDF instrumentation or probe specifications, the position of the test probe, motion artifact noise, and environmental factors of the measurements might affect the acquisition of data [19]. In this study, a unified detection system was established in the preliminary experiments. In addition, the teeth and gums were covered using an opaque silicone rubber impression to reduce the impact of blood flow in the adjacent soft tissues [9]. Changing the probe position on the tooth might significantly affect the output signals from the probe and the reliability of the testing method [22]. For consistency of the measurement positioning on the teeth, the labial surface of the crown was divided into nine equal segments and the central segment that was located slightly cervical to the roof of the pulp chamber and away from the soft tissues was used for probe placement. The external noise, distraction, and displacement of the instrumental probe during measurements could generate experimental errors. To overcome this and minimize involuntary movements, the calibrated laser probe was stabilized to ensure that the measurement position did not change during repeated measurements. The average values that were recorded for 10 s with stable and no obvious amplitude were used as the final measurement data. The present study has certain limitations. For instance, only permanent maxillary incisors from a certain age group (7–12 years) were included; therefore, these findings cannot be generalized for multi-rooted teeth (e.g., molars and premolars) and age groups. Therefore, more clinical studies are required to verify these findings. Some research showed that LDF had certain limitation in the elderly population. According to the reduced pulp cavity volume and blood flow velocity in the elderly, the LDF is not considered to be suitable to determine the age-related changes in the pulp cavity in elderly patients [20]. However, the exact age limit remains unclear. Considering the age-related changes in the root canal, the clinical reference of PBF in more relevant age groups should be obtained in future studies to confirm these conclusions. In addition, the application of LDF for the detection of dental pulp vitality could be explored to determine its use advantages in children's oral diagnosis and treatment.

## Conclusions

LDF could be used to detect the vitality of dental pulp after trauma. The determination of the PBF clinical reference range and clinical coincidence rate for the permanent maxillary incisors in children using LDF provided a promising theoretical basis for clinical applications.

## Data Availability

The datasets used and/or analysed during the current study are available from the corresponding author upon reasonable request.

## Abbreviations

LDF: Laser doppler flowmetry
PBF: Pulp blood flow
